# [Retracted] Metformin-induced activation of AMPK inhibits the proliferation and migration of human aortic smooth muscle cells through upregulation of p53 and IFI16

**DOI:** 10.3892/ijmm.2026.5838

**Published:** 2026-04-22

**Authors:** Biao Hao, Yan Xiao, Fang Song, Xiangshu Long, Jing Huang, Maobo Tian, Shiyan Deng, Qiang Wu

Int J Mol Med 41: 1365-1376, 2018; DOI: 10.3892/ijmm.2017.3346

Following the publication of the above article, a concerned reader drew the Editor's attention to the fact that the figures presented in this paper appeared to contain the following anomalies: In Fig. 4D, the 'Met 5 mM' and 'Met 10 mM' data panels were overlapping; in Fig. 8F, the 'IF116 si' and 'IF116 si + Met' data panels were overlapping; in Fig 5A, the 'Un' and 'Ctr' rows of data were overlapping; and in Fig. 7A, the 'Ctr' and 'IF116 Si' rows of data were overlapping. In addition, after having performed an independent analysis of the data in this paper in the Editorial office, it came to light that the 'Met 0 mM' panel in Fig. 4D contained an overlapping section with the Control panel in Fig. 8F.

Given that these issues have come to light, the Editor of *International Journal of Molecular Medicine* has decided that this article should be retracted from the publication on the grounds of an overall lack of confidence in the presented data. The authors were asked for an explanation to account for these concerns, but the Editorial Office did not receive a satisfactory reply. The Editor sincerely apologizes to the readership for any incovenience caused, and we thank the reader for drawing this matter to our attention.

